# First Report of Circulating MicroRNAs in Tumour Necrosis Factor Receptor-Associated Periodic Syndrome (TRAPS)

**DOI:** 10.1371/journal.pone.0073443

**Published:** 2013-09-16

**Authors:** Orso Maria Lucherini, Laura Obici, Manuela Ferracin, Valerio Fulci, Michael F. McDermott, Giampaolo Merlini, Isabella Muscari, Flora Magnotti, Laura J. Dickie, Mauro Galeazzi, Massimo Negrini, Cosima Tatiana Baldari, Rolando Cimaz, Luca Cantarini

**Affiliations:** 1 Research Center of Systemic Autoimmune and Autoinflammatory Diseases, Rheumatology Unit, Policlinico Le Scotte, University of Siena, Siena, Italy; 2 Amyloid Research and Treatment Center, Fondazione IRCCS Policlinico San Matteo, and Department of Molecular Medicine, University of Pavia, Pavia, Italy; 3 Department of Morphology, Surgery and Experimental Medicine, Pathology Section and Laboratory for Technologies of Advanced Therapies (LTTA), University of Ferrara, Ferrara, Italy; 4 Dipartimento di Biotecnologie Cellulari ed Ematologia, Sezione di Genetica Molecolare, Sapienza Università di Roma, Rome, Italy; 5 NIHR-Leeds Musculoskeletal Biomedical Research Unit (NIHR-LMBRU), Institute of Rheumatic and Musculoskeletal Medicine (LIRMM), Leeds, United Kingdom; 6 Department of Life Sciences, University of Siena, Siena, Italy; 7 Department of Pediatrics, Rheumatology Unit, Anna Meyer Children’s Hospital and University of Florence, Florence, Italy; South Texas Veterans Health Care System and University Health Science Center San Antonio, United States of America

## Abstract

Tumor necrosis factor-receptor associated periodic syndrome (TRAPS) is a rare autosomal dominant autoinflammatory disorder characterized by recurrent episodes of long-lasting fever and inflammation in different regions of the body, such as the musculo-skeletal system, skin, gastrointestinal tract, serosal membranes and eye. Our aims were to evaluate circulating microRNAs (miRNAs) levels in patients with TRAPS, in comparison to controls without inflammatory diseases, and to correlate their levels with parameters of disease activity and/or disease severity. Expression levels of circulating miRNAs were measured by Agilent microarrays in 29 serum samples from 15 TRAPS patients carrying mutations known to be associated with high disease penetrance and from 8 controls without inflammatory diseases. Differentially expressed and clinically relevant miRNAs were detected using GeneSpring GX software. We identified a 6 miRNAs signature able to discriminate TRAPS from controls. Moreover, 4 miRNAs were differentially expressed between patients treated with the interleukin (IL)-1 receptor antagonist, anakinra, and untreated patients. Of these, miR-92a-3p and miR-150-3p expression was found to be significantly reduced in untreated patients, while their expression levels were similar to controls in samples obtained during anakinra treatment. MiR-92b levels were inversely correlated with the number of fever attacks/year during the 1^st^ year from the index attack of TRAPS, while miR-377-5p levels were positively correlated with serum amyloid A (SAA) circulating levels. Our data suggest that serum miRNA levels show a baseline pattern in TRAPS, and may serve as potential markers of response to therapeutic intervention.

## Introduction

Tumor necrosis factor-receptor associated periodic syndrome (TRAPS) is the most common autosomal dominant autoinflammatory disorder and is caused by mutations in the *TNFRSF1A* gene (12p13) encoding the 55-kD receptor for tumor necrosis factor-α (TNF-α) (TNFRSF1A) [1]. TRAPS is characterized by recurrent fever attacks lasting typically from 1 to 3 weeks; in addition to fever, common clinical features include mainly periorbital oedema, conjunctivitis, a migratory erythematous skin rash with underlying fasciitis and myalgia, and arthralgia and/or arthritis [2], [3]; serosal inflammation is also common, usually but not only in the form of polyserositis [4]–[8]. Mean age at disease onset is around 3 years. Nevertheless TRAPS is the most variable and multiform entity amongst autoinflammatory diseases both in terms of age at disease onset and clinical manifestations [2]–[4], [9]. This heterogeneity is probably related to the wide spectrum of known *TNFRSF1A* mutations [10]. TRAPS mutations can be distinguished into high-penetrance variants and low-penetrance variants: the former are mostly missense substitutions, mainly affecting the highly conserved cysteine residues of the extracellular cysteine-rich domains involved in disulfide bond formation and in the folding of the extracellular portion of TNFRSF1A [2], [3]. These mutations are associated with an earlier disease onset and with a more severe phenotype; in fact patients may experience a higher number of fever episodes and a greater severity of attacks [11]. These subjects also have a greater risk of developing AA amyloidosis, the most troublesome TRAPS complication [2], [3], [12]. On the contrary low-penetrance variants seem to be associated with a milder phenotype, a later disease onset and a lower risk of amyloidosis [3]–[9], [13].

The identification of *TNFRSF1A* mutations as the genetic cause of TRAPS raised the possibility that blocking TNF - even though TNF is not increased in most patients –could potentially represent a tailored therapeutic strategy, opening the way to new treatment opportunities for this complex disease [14]. Etanercept has been shown to control flares and inflammation in short case-series of patients of different ages with fully penetrant TRAPS phenotypes and in a prospective, open-label study [15], in which it proved to decrease the frequency of the attacks and the disease severity [16]–[18]. However, loss of response to etanercept over time as well as etanercept-resistant patients have also been observed, suggesting a non-specific action of etanercept in TRAPS [3], [19], [20].

Evidence of deregulated secretion of interleukin (IL)-1β recently supported IL-1 inhibition as a target therapy for TRAPS and IL-1 inhibitors, such as the human IgG1 anti-IL-1β monoclonal antibody canakinumab and the IL-1 receptor antagonist anakinra, have shown to induce a prompt and complete disease remission [21]–[25].

MicroRNAs (miRNAs) are small, non-coding RNAs (∼18–25 nucleotides in length) that regulate gene expression at a post-transcriptional level, by degrading mRNA molecules or blocking their translation [26]. It is now well known that miRNAs can regulate every aspect of cellular activity, from differentiation and proliferation to apoptosis and that, as a single miRNA can target hundreds of mRNAs, aberrant miRNA expression is involved in the pathogenesis of many diseases [27]. These molecules can be detected in serum, and their circulating levels have already been described in inflammatory disorders such as rheumatoid arthritis and systemic lupus erythematosus [28]–[30]. To the best of our knowledge circulating miRNAs in TRAPS, as well as in other monogenic autoinflammatory disorders have never been investigated.

The aim of our study was to evaluate circulating miRNAs levels in patients with TRAPS, in comparison to controls without inflammatory diseases, and to correlate their levels with parameters of disease activity and/or disease severity.

## Materials and Methods

### Patients

Twenty-nine serum samples were obtained from 15 TRAPS patients (8 males, 7 females) carrying mutations known to be associated with high disease penetrance (C43R 2/15 pts; C43Y 1/15 pts; T50M 2/15 pts; C52Y 3/15 pts; C55Y 1/15 pts; S59N 1/15 pts; S59P 1/15 pts; C88Y 1/15 pts; Del 103–104 1/15 pts; C114W: 1/15 pts; L167–175del: 1/15 pts). Patients were evaluated between January 2008 and November 2012 at our institutions. At least one sample was obtained for each patient in the absence of any treatment. For 6 patients more than one sample was obtained at different time points in the absence of any treatment, resulting in a total of 22 samples collected in untreated individuals. Seven samples were obtained from different patients during anakinra therapy.

Samples were also obtained from 8 age- and gender-matched controls without inflammatory diseases (5 males, 3 females) (41 years; range 21–59) attending the outpatient clinic at the Rheumatology Unit of the University of Siena, for fibromyalgia and/or carpal tunnel syndrome and who tested negative for TRAPS mutations. These subjects underwent detailed clinical and laboratory workup, in order to rule out any inflammatory, metabolic, and neoplastic disorders (in particular, they all showed inflammatory markers within normal values). Table 1 summarises the clinical and demographic characteristics of the samples collected from treated and untreated TRAPS patients. All patients and controls were Caucasians of Italian origin. Written informed consent was obtained both from patients and controls. The study protocol was reviewed and approved by the University of Siena Institutional Ethics Committee.

**Table 1:** The table summarises clinical and demographic characteristics of TRAPS patients treated with anakinra versus untreated patients (at the time of sample collection), expressed as median (range) when necessary.

	Samples from treated patients (n = 7)	Samples from untreated patients (n = 22)
Age (yrs)	41 (31–52)	42 (19–69)
Disease onset (yrs)	4 (1–26)	4 (1–49)
N° of fever attacks/year	4 (2–10)	4 (2–10)
Duration of fever attacks (days)	9 (0–10)	10 (0–15)
ESR (mm/h)	5 (5–82)	50 (10–90)
CRP (mg/dl)	0 (0.1–5.14)	5 (0.1–16.1)
SAA (mg/l)	7 (3.5–202)	173 (2–1510)

**List of abbreviations**: TRAPS, tumor necrosis factor receptor-associated periodic syndrome; ESR, erythrocyte sedimentation rate; CRP, C-reactive protein; SAA, serum amyloid A; F, female; M, male; yrs, years; Y, yes; N, no.

### Methods

Data collected in a customized database for each subject with TRAPS included: i) gender ii) age iii) age at disease onset iv) disease duration v) duration of fever episodes vi) number of fever episodes/year (at disease onset) vii) number of fever episodes/year (during the last year) viii) amyloidosis (presence/absence) ix) chronic disease course (presence/absence) x) treatment with IL-1 inhibiting drugs (yes/no).

### Laboratory Assessment

Blood was taken by venipuncture from patients during routine follow-up. Laboratory assessment included erythrocyte sedimentation rate (ESR), C-reactive protein (CRP), and SAA. ESR was measured using the Westergren method (mm/hour), and was considered normal if <15 mm/hour for males and <20 mm/hour for females. Serum CRP concentrations were measured using a nephelometric immunoassay (mg/dl); values <0.5 mg/dl were considered normal. Serum amyloid A (SAA) levels were measured by particle-enhanced nephelometry (BNII autoanalyzer, Dade Behring, Marburg, Germany). Reference value is <6.4 mg/L.

### RNA Extraction

Blood was centrifuged after venipuncture, and serum was immediately frozen at −80°C until assayed. Total RNA including microRNAs was extracted from 200 µl of serum using miRNeasy Mini Kit (cat. no. 217004 Qiagen) according to the manufacturer’s supplementary protocol with minimal variations. Specifically, 2.5 µl of 5 nM synthetic miRNAs cel-miR-39, cel-miR-54, cel-miR-238, ebv-miR-BART8, hcmv-miR-UL112, kshv-miR-K12-2 (IDT) were added immediately after QIAzol Lysis Reagent. RNA was eluted in 35 µl and 10 µl were used for microarray hybridization.

### miRNA Expression Profiling

Thirty-seven RNA samples, from 8 controls (8 samples) and 15 TRAPS patients (29 samples), were hybridized on Agilent human miRNA microarray (#G4470B, Agilent Technologies, Palo Alto, CA). This chip consists of 15,000 probes, which represent 723 human microRNAs, sourced from the Sanger miRBase database (Release 10.1). Starting from equal volumes, RNA labeling and hybridization were performed in accordance to manufacturer’s indications, as we previously reported [31]. Agilent scanner and the Feature Extraction 10.5 software (Agilent Technologies) were used to obtain the microarray raw-data.

Microarray results were analysed using the GeneSpring GX 12 software (Agilent Technologies, Palo Alto, CA). Data transformation was applied to set all negative raw values at 1.0, followed by normalization on spiked-in control viral miRNAs (ebv-miR-BART8, hcmv-miR-UL112, kshv-miR-K12-2). A filter on low gene expression was used to keep only the probes expressed in at least one sample. Differentially expressed genes were selected to have a 1.5-or 2-fold expression difference between groups and a statistically significant p-value (<0.05), using moderated t-test with or without Benjamini-Hochberg correction, as indicated in the text. Differentially expressed genes were employed for Cluster Analysis of samples using the Manhattan correlation as a measure of similarity.

### Statistical Analysis

Correlation analysis between all miRNAs and clinical variables was performed using PAM (Prediction Analysis of Microarrays) software [32]. Statistical analyses were performed using GraphPad Prism 5 software. Two-tailed Mann-Whitney test was used for statistical comparisons between groups. Correlations were calculated using log2 transformed data and Spearman’s correlation (two-tailed p-value).

## Results

**Figure 1:**
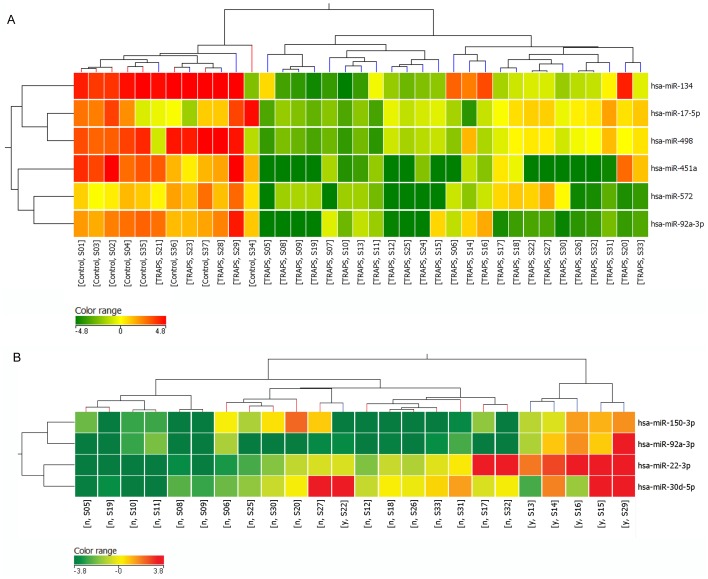
Hierarchical cluster representation of miRNAs modulated in TRAPS. Cluster analysis groups samples and genes according to expression similarity. Genes are in rows, samples in columns. The colors of the genes represented on the heatmap correspond to the values normalized on miRNA average expression across all samples (see legends); up-regulated miRNAs are in red, down-regulated miRNAs in green. **A)** Cluster analysis of miRNAs modulated in TRAPS (blue) vs. controls (red) **B)** Four miRNAs are differentially expressed in samples from TRAPS patients during treatment with the interleukin (IL)-1 receptor antagonist anakinra. Treated patients (y) are colored in blue, whereas untreated patients (n) in red. One treated patient (S22) displays and intermediate expression profile.

Microarray analysis performed on TRAPS patients and controls revealed 172 miRNAs whose expression was detectable in at least 1 sample (Table S1). The TRAPS miRNA expression profile was compared with that of controls, revealing a signature of six miRNAs specific for TRAPS patients (corrected p-value <0.05, Table 2), which were all significantly down-regulated in TRAPS patients (miR-17-5p, miR-92a-3p, miR-134, miR-451a, miR-498, miR-572,) and were used for cluster analysis (Figure 1A). The exclusion of anakinra treated samples increased the number of differentially expressed miRNAs by two additional units, namely miR-150-3p and miR-187-5p (Table S2). Figure S1 represents the hierarchical cluster of untreated TRAPS patients and controls based on this gene signature.

**Table 2:** MiRNAs significantly modulated in TRAPS patients versus controls.

microRNA	p (Corr)	p	Fold change	Regulation	Chromosome	miRBase accession number
hsa-miR-17-5p	0.012103879	4.93E-04	6.7467217	down	chr13	MIMAT0000070
hsa-miR-134	0.02169203	0.002648446	16.012423	down	chr14	MIMAT0000447
hsa-miR-92a-3p	0.012103879	3.41E-04	22.467995	down	chr13	MIMAT0000092
hsa-miR-451a	0.009554302	1.51E-04	81.65974	down	chr17	MIMAT0001631
hsa-miR-498	0.009554302	1.67E-04	14.842656	down	chr19	MIMAT0002824
hsa-miR-572	0.025193842	0.003376849	9.518456	down	chr4	MIMAT0003237

This signature distinguished between the TRAPS patients and control group, with only three TRAPS samples (2 samples with T50M and one with S59P) displaying a profile similar to controls.

To evaluate possible miRNA modulation in TRAPS subgroups, we examined the effect of treatment with the IL-1 receptor antagonist anakinra on circulating miRNA profiles, by comparing treated versus untreated patients. We found 4 miRNAs whose expression was significantly altered after anakinra treatment (p-value <0.05, Table 3).

**Table 3:** MiRNAs differentially expressed between treated and untreated TRAPS patients.

microRNA	p	Fold change	Regulation	Chromosome	mirbase accession
hsa-miR-22-3p	4.79E-05	30.181482	up	chr17	MIMAT0000077
hsa-miR-30d-5p	0.003982957	11.236352	up	chr8	MIMAT0000245
hsa-miR-92a-3p*	3.88E-06	28.059376	up	chr13	MIMAT0000092
hsa-miR-150-3p*	0.017138826	6.3681154	up	chr19	MIMAT0004610

* These microRNAs are downregulated in TRAPS patients vs. controls (see Table 2 and Table S2).

Figure 1B represents the cluster analysis of TRAPS samples based on the expression of 4 miRNAs that differentiate treated from untreated patients. We obtained a very good separation between the two groups; indeed only one sample was misclassified.

**Figure 2:**
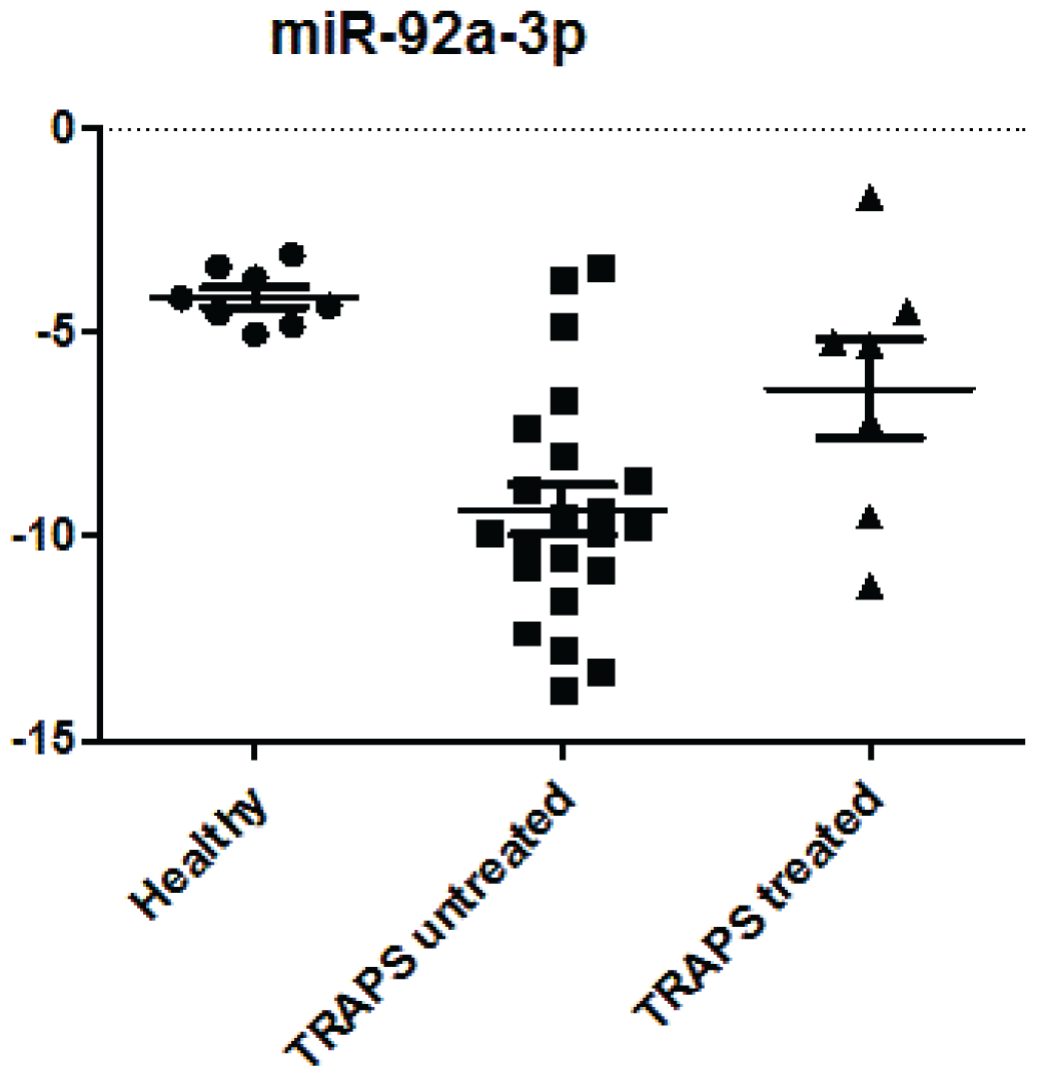
Expression of miR-92-3p and miR-150-3p in controls and in treated and untreated TRAPS patients. The expression levels of miR-93-3p and miR-150-3p, derived from the microarray experiment, were plotted in this graph using scatter plot distribution. ** p-value <0.001 at t-test; * p-value <0.05 at t-test.

Interestingly, miR-92a-3p and miR-150-3p expression levels were found to be significantly reduced in untreated TRAPS patients, while their expression levels were similar to controls in those patients who were sampled during treatment (Figure 2).

**Figure 3:**
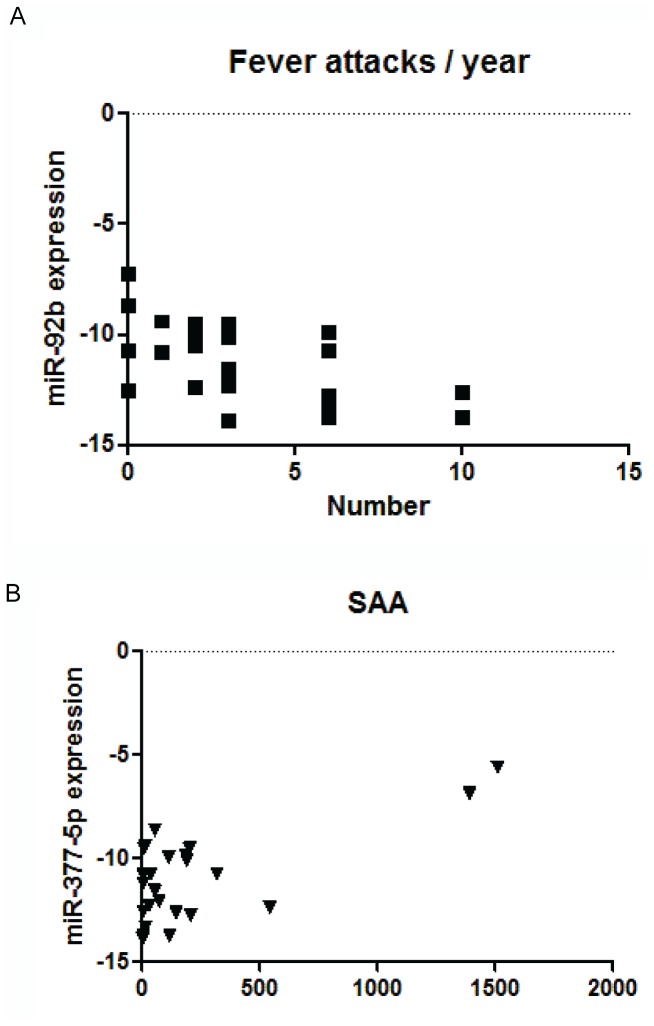
MicroRNAs correlating with clinical and laboratory features. **A)** correlation of miR-92b with the number of fever attacks/year during the 1^st^ year from the index attack of TRAPS. The levels of miR-92b correlate negatively with the number of fever attacks/year during the 1^st^ year from the index attack of TRAPS (p 0.0045, r = −0.5589) **B)** the levels of miR-377-5p are increased in patients presenting increased serum amyloid A (SAA) levels (p 0.0004, r = 0.66).

Finally we searched for possible correlations between miRNA signatures and the clinical and laboratory variables reflecting disease severity and/or disease activity. MiR-92b levels were inversely correlated with the number of fever attacks/year during the 1^st^ year from the index attack of TRAPS (Spearman r = −0.5589) (Figure 3A), while miR-377-5p levels were positively correlated with SAA circulating levels (Spearman r = 0.66) (Figure 3B **)**.

## Discussion

In recent years, scientific interest in miRNAs has increased dramatically since they have been shown to play a key role in the regulation of immunity, including innate and adaptive immune responses, development, and differentiation of immune cells [33]. Abnormal miRNA expression has also been reported in rheumatic autoimmune diseases in which it has been observed that miRNAs can be variably expressed according to disease activity and progression [30], [34], [35]. Altogether, information available to date points to miRNAs as potential biomarkers, which could be exploited to monitor disease activity and response to drugs [35]. MiRNAs themselves are also emerging as potential targets for new therapeutic strategies. The ultimate goal will be the identification of a miRNA target or targets that could be manipulated through specific therapies, aiming at activation or inhibition of specific miRNAs responsible for the development of disease [28].

Lawrie et al. were among the first to demonstrate the presence of circulating miRNAs in cell-free body fluids such as plasma and serum [36]. MiRNAs have been reported as being aberrantly expressed in blood plasma or serum in different types of disorders such as cancer, diabetes mellitus and cardiovascular diseases [36]–[38]; moreover, changes in circulating miRNAs profiling have shown potential diagnostic as well as prognostic value in several disorders [39]. The exact mechanism by which miRNAs enter into the serum and whether they are biologically functional or simply biomarkers is still unknown. A recent study reported that miRNAs could be selectively packaged into micro-vesicles and actively secreted [40]. To the best of our knowledge no information concerning circulating miRNAs in TRAPS is available.

Our data show that circulating miRNAs levels are altered in TRAPS patients versus controls. Six miRNAs were found to be downregulated in TRAPS (miR-134, miR-17-5p, miR-498, miR-451a, miR-572, miR-92a-3p) in comparison with controls. Moreover the expression of 4 additional miRNAs (miR-150-3p, miR-92a-3p, miR-22-3p, miR-30d-5p) was significantly altered in untreated patients versus subjects treated with anakinra. It is worthy of notice that miR-92a-3p and miR-150-3p levels, which are reduced in untreated TRAPS patients compared with controls, are restored to levels comparable with controls during anakinra treatment. In addition, the expression of other specific circulating miRNAs significantly correlated with both the number of fever attacks/year at disease onset (miR-92b levels were significantly reduced), suggesting a relationship with disease severity, and with enhanced SAA levels (miR-377-5p levels were significantly enhanced). Among the miRNAs which we found to be altered in TRAPS, MiR-150, miR-92 and miR-17 have been shown to be key regulators of specific lineage choices in the adaptive immune system and serum miR-150 levels have recently been negatively correlated with the plasma TNF-α levels in patients with sepsis [41]. Their role in TRAPS still needs to be elucidated.

A growing number of studies have shown that circulating miRNA expression profiling is of increasing importance as a useful diagnostic and prognostic tool. In patients with a TRAPS-like disease, characterized by clinical features consistent with TRAPS but no mutation in the *TNFRSF1A* gene [42], circulating miRNA expression profiling could potentially become useful as a diagnostic signature, opening the way to a better understanding of the molecular mechanisms underlying this complex phenotype, Moreover, our data suggest that serum miRNA levels could be taken into future consideration for monitoring the response to treatment in TRAPS patients. Looking a bit further into the future, a better knowledge about miRNAs role in TRAPS could also potentially provide novel treatment targets [40], [43]–[46]. We acknowledge the limitations of our small samples size, and therefore a further study involving larger cohorts of patients and the use of different technical approaches is being planned.

## Conclusions

Although further studies are mandatory, serum miRNAs levels show a baseline pattern in TRAPS and thus deserve further studies to exploit their potential role as markers of disease and response to therapeutic intervention.

## Supporting Information

Figure S1 **Hierarchical cluster representation of miRNAs modulated in untreated TRAPS patients (blue) vs. controls (red).**
(TIF)

Table S1 **Normalized and log2 transformed expression levels of 172 miRNAs whose expression was detectable in at least 1 sample.**
(XLSX)

Table S2 **MiRNAs significantly modulated in untreated TRAPS patients versus controls. Expression in treated TRAPS patients is also reported.**
(XLSX)
