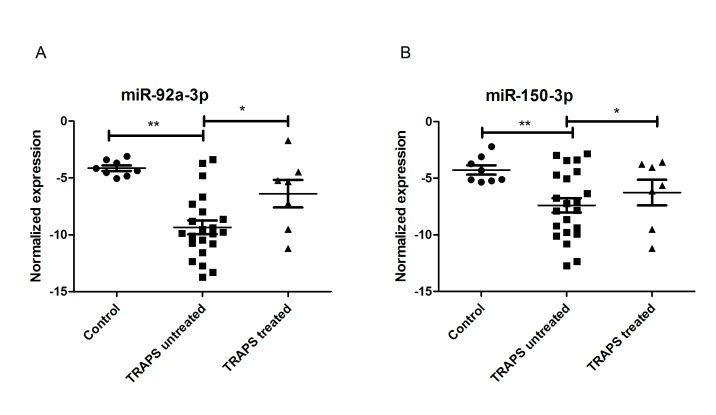# Correction: First Report of Circulating MicroRNAs in Tumour Necrosis Factor Receptor-Associated Periodic Syndrome (TRAPS)

**DOI:** 10.1371/annotation/e0c975da-3b24-4e55-b63d-296986b90c0b

**Published:** 2013-10-10

**Authors:** Orso Maria Lucherini, Laura Obici, Manuela Ferracin, Valerio Fulci, Michael F. McDermott, Giampaolo Merlini, Isabella Muscari, Flora Magnotti, Laura J. Dickie, Mauro Galeazzi, Massimo Negrini, Cosima Tatiana Baldari, Rolando Cimaz, Luca Cantarini

Due to an error in the production process, the version of Figure 2 that appeared in the article was incorrect. The correct version is available here: 

**Figure:**