# Supplementary figures and images for: First Report of Circulating MicroRNAs in Tumour Necrosis Factor Receptor-Associated Periodic Syndrome (TRAPS)
Source: PLoS One. 2013 Sep 16;8(9):e73443. doi: 10.1371/journal.pone.0073443 (PMC3774691; doi:10.1371/journal.pone.0073443)

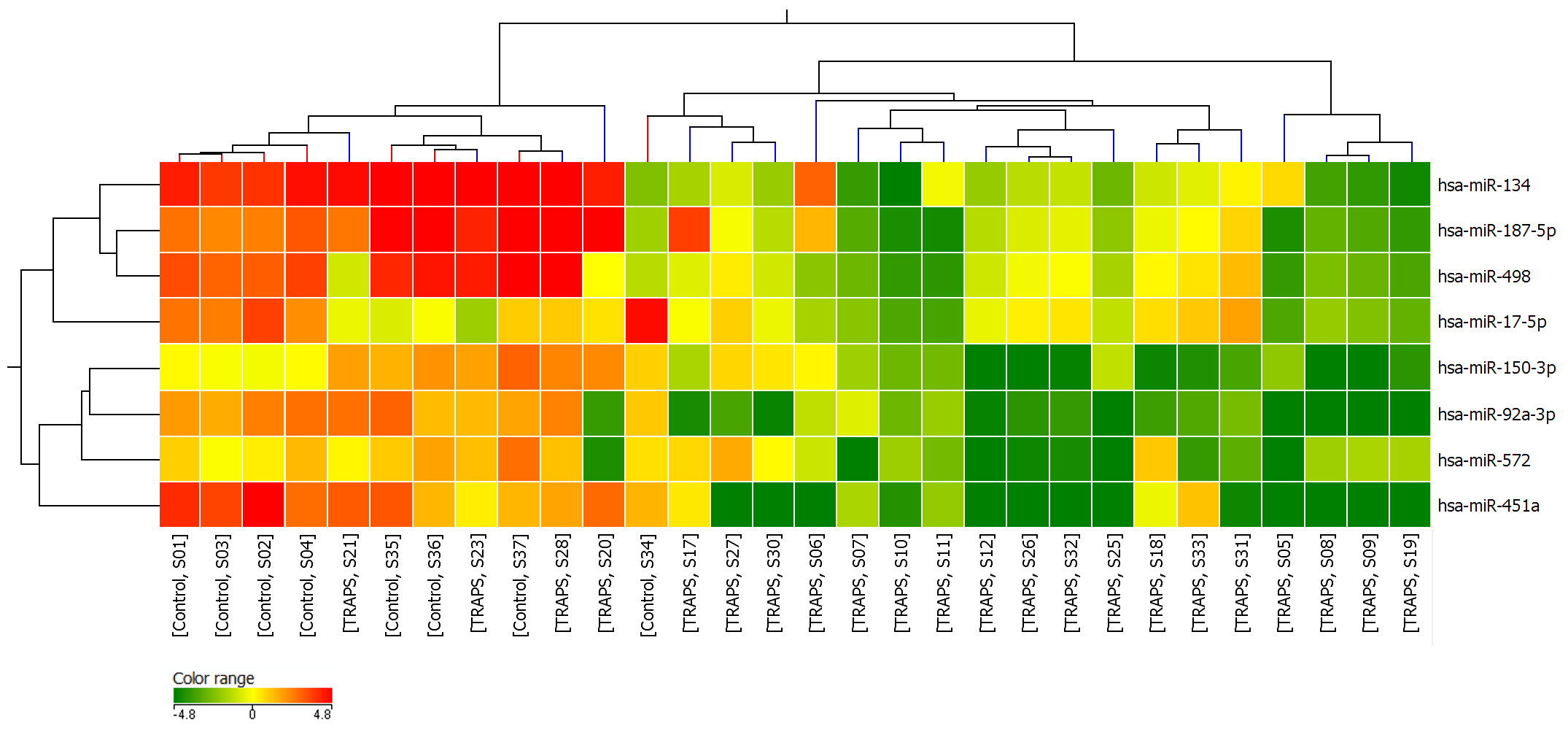

Supplement: Figure S1 — Hierarchical cluster representation of miRNAs modulated in untreated TRAPS patients (blue) vs. controls (red). (TIF) [file pone.0073443.s001.tif]
